# 4-(4-Methyl­phen­yl)-2-(prop-2-yn-1-yl)phthalazin-1(2*H*)-one

**DOI:** 10.1107/S1600536813034880

**Published:** 2014-01-15

**Authors:** M. K. Usha, S. Madan Kumar, P. C. Shyma, B. Kalluraya, N. K. Lokanath, D. Revannasiddaiah

**Affiliations:** a Department of Studies in Physics, University of Mysore, Manasagangotri, Mysore 570 006, India; b Department of Studies in Chemistry, Mangalore University, Mangalagangotri, Mangalore 574 199, India

## Abstract

In the title compound, C_18_H_14_N_2_O, the dihedral angle between the methyl­phenyl ring and the phthalazone ring system (r.m.s. deviation = 0.034 Å) is 53.93 (9)°. In the crystal, mol­ecules are connected by C—H⋯O hydrogen bonds, forming chains along [101]. The chains are linked by π–π inter­actions [centroid–centroid distance 3.6990 (12) Å], forming layers parallel to (10-1).

## Related literature   

For general background and the biological and pharmacological properties of phthalazine derivatives, see: Abd alla *et al.* (2010 ▶); Awadallah *et al.* (2012 ▶); Khalil *et al.* (2009 ▶); Kim *et al.* (2008 ▶); Ryu *et al.* (2007 ▶). For a related structure, see: Bausch *et al.* (1997 ▶).
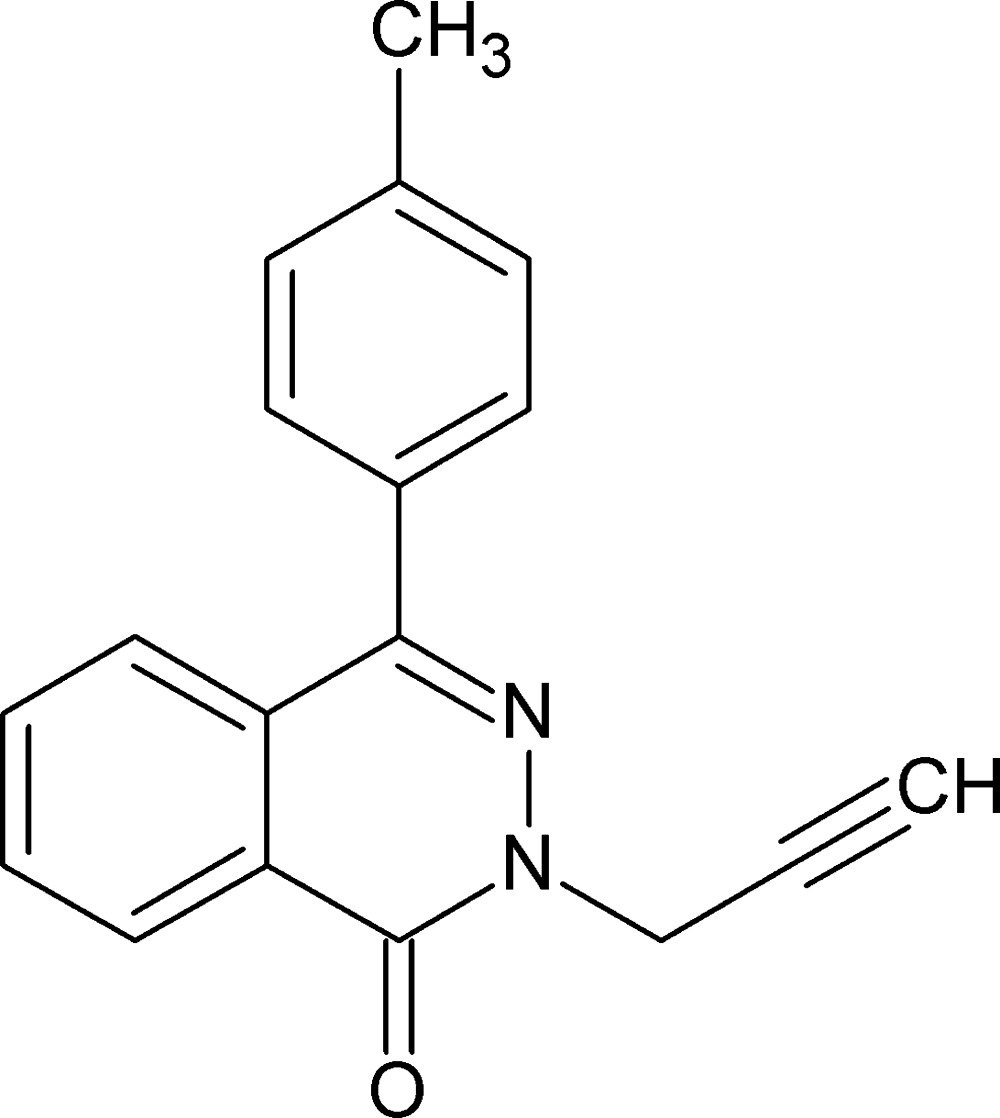


## Experimental   

### 

#### Crystal data  

C_18_H_14_N_2_O
*M* *_r_* = 274.31
Monoclinic, 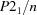

*a* = 11.9917 (19) Å
*b* = 9.7116 (16) Å
*c* = 12.602 (2) Å
β = 101.285 (7)°
*V* = 1439.2 (4) Å^3^
*Z* = 4
Cu *K*α radiation
μ = 0.63 mm^−1^
*T* = 296 K
0.23 × 0.20 × 0.19 mm

#### Data collection  

Bruker X8 Proteum diffractometer
Absorption correction: multi-scan (*SADABS*; Bruker, 2013 ▶) *T*_min_ = 0.864, *T*_max_ = 0.886
8733 measured reflections
2337 independent reflections
2093 reflections with *I* > 2σ(*I*)
*R*_int_ = 0.036

#### Refinement  

*R*[*F* ^2^ > 2σ(*F* ^2^)] = 0.044
*wR*(*F* ^2^) = 0.132
*S* = 1.10
2337 reflections
192 parameters
H-atom parameters constrained
Δρ_max_ = 0.15 e Å^−3^
Δρ_min_ = −0.15 e Å^−3^

### 

Data collection: *APEX2* (Bruker, 2013 ▶); cell refinement: *SAINT* (Bruker, 2013 ▶); data reduction: *SAINT*; program(s) used to solve structure: *SHELXS97* (Sheldrick, 2008 ▶); program(s) used to refine structure: *SHELXL97* (Sheldrick, 2008 ▶); molecular graphics: *Mercury* (Macrae *et al.*, 2008 ▶); software used to prepare material for publication: *PLATON* (Spek, 2009 ▶).

## Supplementary Material

Crystal structure: contains datablock(s) global, I. DOI: 10.1107/S1600536813034880/is5329sup1.cif

Structure factors: contains datablock(s) I. DOI: 10.1107/S1600536813034880/is5329Isup2.hkl

Supporting information file. DOI: 10.1107/S1600536813034880/is5329Isup3.cml

CCDC reference:

Additional supporting information:  crystallographic information; 3D view; checkCIF report

## Figures and Tables

**Table 1:** Hydrogen-bond geometry (Å, °)

*D*—H⋯*A*	*D*—H	H⋯*A*	*D*⋯*A*	*D*—H⋯*A*
C6—H6⋯O1^i^	0.93	2.45	3.322 (2)	157

Symmetry code: (i) 
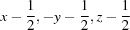
.

**Fig. 1.**
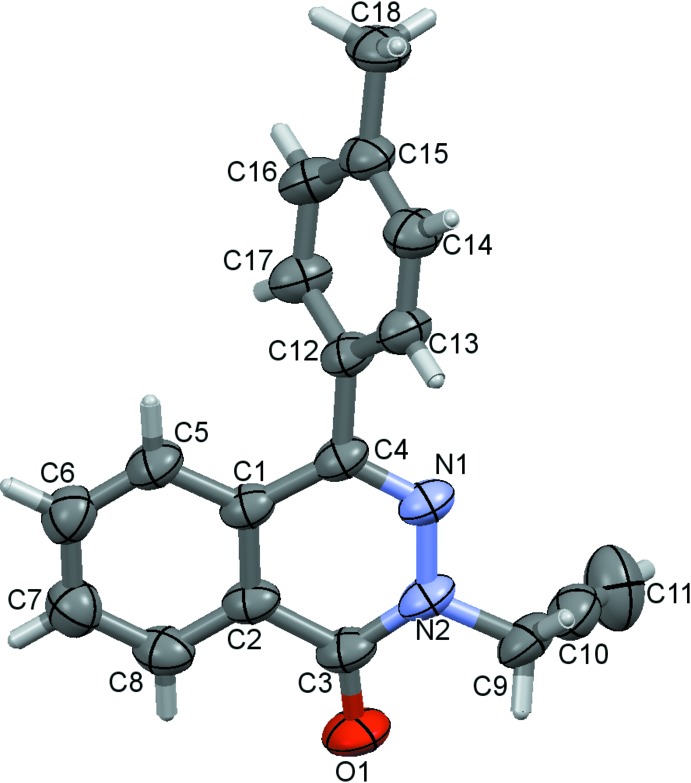
ORTEP diagram of the title compound with 50% probability ellipsoids.

**Fig. 2.**
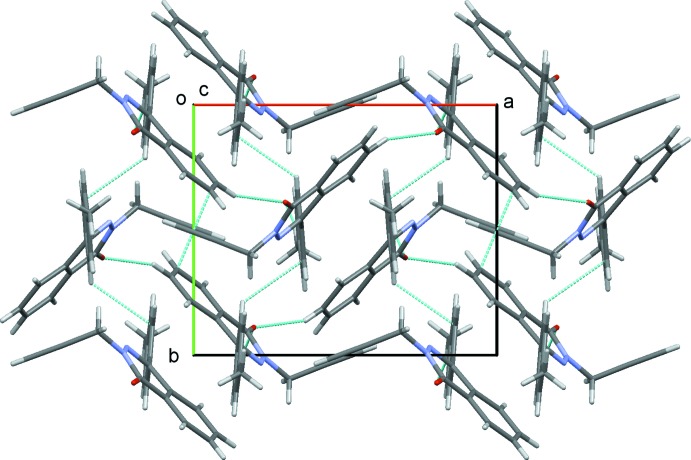
Packing diagram of the title compound, viewed along the c axis.

## supplementary crystallographic information

### 1. Comment

Nitrogen heterocyclic compounds have received a great attention because of their wide applicability in different areas, especially drugs (Kim *et al.*, 2008). Phthalazines are an important class of nitrogen heterocyclic compounds that possess exciting pharmacological and biological properties (Khalil *et al.*, 2009). Phthalazines have been reported to possess antifungal (Ryu *et al.*, 2007), antibacterial (Khalil *et al.*, 2009), cytotoxic (Kim *et al.*, 2008), anti-inflammatory (Abd alla *et al.*, 2010), antihypertensive and vasorelaxant (Awadallah *et al.*, 2012) properties. As part of our studies in this area, herewith we report the structure of the title compound.

The *ORTEP* of the title compound is shown (Fig. 1). The phthalazine ring is nearly planar. The dihedral angle between the methylphenyl ring and the phenyl ring of the phthalazinone moiety is 53.93 (9)°. The bond lengths and bond angles of the title compound are comparable to related structure, 4-(9-fluorenoxy)-2-phenylphthalazin-1(*2H*)-one (Bausch *et al.*, 1997). In the crystal structure, molecules are connected by intermolecular C—H···O hydrogen bonds (Fig. 2). Also, short contacts of the type π–π are observed [minimum centroid-centroid distance 3.6990 (12) Å].

### 2. Experimental

2-(4-Methylbenzoyl)benzoic acid (0.1 mol) is esterified in ethanol (25 ml) in presence of few drops of sulfuric acid. The ethyl 2-(4-methylbenzoyl)benzoate obtained is further refluxed with hydrazine hydrate (5 ml, 98%) in absolute ethanol (50 ml)for 2 hrs. Solid obtained on cooling was filtered off and dried to give 4-(4-methylphenyl)phthalazin-1-ol*. A* mixture of 4-(4-methylphenyl)phthalazin-1-ol (0.015 mol), anhydrous potassium carbonate (3.04 g, 0.022 mol) and propargylbromide (1.78 g, 0.015 mol) in DMF (25 ml) was stirred at 65 °C for 2 h. After completion of reaction, reaction mixture was poured into ice-cold water. The solid product obtained was purified by column chromatography using n-hexane and ethyl acetate as eluent to get pure compound. Further the compound was recrystallized from ethyl acetate to get the yellow crystals (m.p. 168–170 °C).

### 3. Refinement

The H atoms were placed in calculated positions (C—H = 0.93–0.97 Å) and refined as riding with *U*_iso_(H) = 1.2*U*_eq_(C) or 1.5*U*_eq_(C_methyl_).

### Crystal data

**Table:**

C_18_H_14_N_2_O	*F*(000) = 576
*M**_r_* = 274.31	*D*_x_ = 1.266 Mg m^−^^3^
Monoclinic, *P*2_1_/*n*	Cu *K*α radiation, λ = 1.54178 Å
Hall symbol: -P 2yn	Cell parameters from 2337 reflections
*a* = 11.9917 (19) Å	θ = 4.7–64.6°
*b* = 9.7116 (16) Å	µ = 0.63 mm^−^^1^
*c* = 12.602 (2) Å	*T* = 296 K
β = 101.285 (7)°	Block, yellow
*V* = 1439.2 (4) Å^3^	0.23 × 0.20 × 0.19 mm
*Z* = 4	

### Data collection

**Table:**

Bruker X8 Proteum diffractometer	2337 independent reflections
Radiation source: Bruker MicroStar microfocus rotating anode	2093 reflections with *I* > 2σ(*I*)
Helios multilayer optics monochromator	*R*_int_ = 0.036
Detector resolution: 10.7 pixels mm^-1^	θ_max_ = 64.6°, θ_min_ = 4.7°
φ and ω scans	*h* = −13→12
Absorption correction: multi-scan (*SADABS*; Bruker, 2013)	*k* = −10→11
*T*_min_ = 0.864, *T*_max_ = 0.886	*l* = −8→14
8733 measured reflections	

### Refinement

**Table:**

Refinement on *F*^2^	Secondary atom site location: difference Fourier map
Least-squares matrix: full	Hydrogen site location: inferred from neighbouring sites
*R*[*F*^2^ > 2σ(*F*^2^)] = 0.044	H-atom parameters constrained
*wR*(*F*^2^) = 0.132	*w* = 1/[σ^2^(*F*_o_^2^) + (0.0698*P*)^2^ + 0.2752*P*] where *P* = (*F*_o_^2^ + 2*F*_c_^2^)/3
*S* = 1.10	(Δ/σ)_max_ = 0.003
2337 reflections	Δρ_max_ = 0.15 e Å^−^^3^
192 parameters	Δρ_min_ = −0.15 e Å^−^^3^
0 restraints	Extinction correction: *SHELXL*, FC^*^=KFC[1+0.001XFC^2^Λ^3^/SIN(2Θ)]^-1/4^
Primary atom site location: structure-invariant direct methods	Extinction coefficient: 0.0090 (9)

### Special details

**Table:**

Geometry. Bond distances, angles *etc*. have been calculated using the rounded fractional coordinates. All su's are estimated from the variances of the (full) variance-covariance matrix. The cell e.s.d.'s are taken into account in the estimation of distances, angles and torsion angles
Refinement. Refinement on *F*^2^ for ALL reflections except those flagged by the user for potential systematic errors. Weighted *R*-factors *wR* and all goodnesses of fit *S* are based on *F*^2^, conventional *R*-factors *R* are based on *F*, with *F* set to zero for negative *F*^2^. The observed criterion of *F*^2^ > σ(*F*^2^) is used only for calculating -*R*-factor-obs *etc*. and is not relevant to the choice of reflections for refinement. *R*-factors based on *F*^2^ are statistically about twice as large as those based on *F*, and *R*-factors based on ALL data will be even larger.

### Fractional atomic coordinates and isotropic or equivalent isotropic displacement parameters (Å^2^)

**Table:**

	*x*	*y*	*z*	*U*_iso_*/*U*_eq_	
O1	0.19809 (12)	−0.11059 (16)	1.21105 (8)	0.0755 (5)	
N1	0.22126 (12)	0.03101 (14)	0.95663 (10)	0.0524 (4)	
N2	0.23069 (12)	0.00807 (15)	1.06549 (10)	0.0548 (5)	
C1	0.07580 (12)	−0.14488 (16)	0.92636 (11)	0.0464 (5)	
C2	0.09215 (13)	−0.17108 (17)	1.03779 (11)	0.0486 (5)	
C3	0.17640 (14)	−0.09248 (18)	1.11276 (12)	0.0536 (5)	
C4	0.14781 (13)	−0.04216 (16)	0.88990 (11)	0.0468 (5)	
C5	−0.01049 (14)	−0.21765 (18)	0.85760 (13)	0.0562 (5)	
C6	−0.07462 (15)	−0.3140 (2)	0.89874 (15)	0.0645 (6)	
C7	−0.05524 (16)	−0.3416 (2)	1.00855 (15)	0.0647 (6)	
C8	0.02715 (15)	−0.27061 (19)	1.07770 (14)	0.0588 (6)	
C9	0.31525 (16)	0.0952 (2)	1.13355 (14)	0.0646 (6)	
C10	0.43037 (18)	0.0402 (2)	1.14605 (14)	0.0653 (7)	
C11	0.5226 (2)	−0.0013 (3)	1.1548 (2)	0.0997 (10)	
C12	0.14520 (13)	−0.01379 (17)	0.77355 (12)	0.0473 (5)	
C13	0.13700 (15)	0.11933 (18)	0.73343 (13)	0.0554 (6)	
C14	0.13974 (16)	0.1456 (2)	0.62603 (13)	0.0604 (6)	
C15	0.15141 (14)	0.0409 (2)	0.55518 (12)	0.0567 (6)	
C16	0.15944 (16)	−0.0918 (2)	0.59542 (13)	0.0638 (6)	
C17	0.15584 (16)	−0.11952 (19)	0.70219 (13)	0.0583 (6)	
C18	0.15649 (17)	0.0690 (2)	0.43701 (13)	0.0708 (7)	
H5	−0.02440	−0.20060	0.78360	0.0670*	
H6	−0.13170	−0.36110	0.85220	0.0770*	
H7	−0.09810	−0.40830	1.03530	0.0780*	
H8	0.03990	−0.28870	1.15150	0.0710*	
H9A	0.31310	0.18630	1.10180	0.0770*	
H9B	0.29570	0.10410	1.20440	0.0770*	
H11	0.59650	−0.03440	1.16180	0.1200*	
H13	0.12950	0.19220	0.77940	0.0660*	
H14	0.13360	0.23590	0.60110	0.0720*	
H16	0.16750	−0.16440	0.54940	0.0760*	
H17	0.16060	−0.21010	0.72650	0.0700*	
H18A	0.12210	0.15650	0.41580	0.1060*	
H18B	0.11620	−0.00210	0.39220	0.1060*	
H18C	0.23440	0.07020	0.42870	0.1060*	

### Atomic displacement parameters (Å^2^)

**Table:**

	*U*^11^	*U*^22^	*U*^33^	*U*^12^	*U*^13^	*U*^23^
O1	0.0829 (9)	0.1064 (11)	0.0305 (6)	−0.0022 (8)	−0.0054 (6)	0.0047 (6)
N1	0.0592 (8)	0.0597 (8)	0.0341 (7)	0.0033 (6)	−0.0011 (6)	−0.0022 (6)
N2	0.0602 (8)	0.0656 (9)	0.0324 (7)	0.0008 (7)	−0.0062 (6)	−0.0062 (6)
C1	0.0452 (8)	0.0546 (9)	0.0356 (8)	0.0106 (7)	−0.0011 (6)	−0.0001 (6)
C2	0.0463 (8)	0.0616 (10)	0.0347 (8)	0.0120 (7)	0.0004 (6)	0.0012 (7)
C3	0.0546 (9)	0.0684 (11)	0.0338 (8)	0.0099 (8)	−0.0010 (7)	−0.0002 (7)
C4	0.0485 (8)	0.0534 (9)	0.0340 (8)	0.0077 (7)	−0.0029 (6)	−0.0022 (6)
C5	0.0558 (9)	0.0676 (11)	0.0398 (8)	0.0020 (8)	−0.0041 (7)	−0.0023 (7)
C6	0.0544 (10)	0.0732 (12)	0.0611 (11)	−0.0037 (9)	−0.0001 (8)	−0.0053 (9)
C7	0.0560 (10)	0.0724 (12)	0.0646 (11)	−0.0008 (9)	0.0089 (8)	0.0059 (9)
C8	0.0555 (9)	0.0757 (12)	0.0446 (9)	0.0087 (8)	0.0084 (7)	0.0102 (8)
C9	0.0735 (12)	0.0688 (12)	0.0429 (9)	−0.0040 (9)	−0.0094 (8)	−0.0117 (8)
C10	0.0669 (12)	0.0716 (12)	0.0519 (10)	−0.0129 (9)	−0.0015 (8)	−0.0003 (8)
C11	0.0672 (15)	0.0930 (17)	0.135 (2)	−0.0067 (13)	0.0102 (14)	0.0109 (15)
C12	0.0463 (8)	0.0578 (9)	0.0338 (8)	0.0043 (7)	−0.0016 (6)	−0.0019 (6)
C13	0.0683 (10)	0.0552 (10)	0.0416 (9)	−0.0007 (8)	0.0081 (7)	−0.0051 (7)
C14	0.0725 (11)	0.0613 (10)	0.0462 (9)	−0.0006 (8)	0.0089 (8)	0.0060 (8)
C15	0.0498 (9)	0.0820 (12)	0.0360 (8)	−0.0015 (8)	0.0027 (7)	−0.0015 (8)
C16	0.0757 (12)	0.0726 (12)	0.0424 (9)	0.0067 (9)	0.0102 (8)	−0.0125 (8)
C17	0.0726 (11)	0.0572 (10)	0.0434 (9)	0.0116 (8)	0.0075 (8)	−0.0021 (7)
C18	0.0664 (11)	0.1084 (16)	0.0362 (9)	−0.0050 (11)	0.0064 (8)	0.0095 (9)

### Geometric parameters (Å, º)

**Table:**

O1—C3	1.2275 (18)	C14—C15	1.378 (3)
N1—N2	1.3725 (18)	C15—C16	1.381 (3)
N1—C4	1.303 (2)	C15—C18	1.527 (2)
N2—C3	1.373 (2)	C16—C17	1.381 (2)
N2—C9	1.463 (2)	C5—H5	0.9300
C1—C2	1.4026 (19)	C6—H6	0.9300
C1—C4	1.452 (2)	C7—H7	0.9300
C1—C5	1.404 (2)	C8—H8	0.9300
C2—C3	1.457 (2)	C9—H9A	0.9700
C2—C8	1.396 (2)	C9—H9B	0.9700
C4—C12	1.486 (2)	C11—H11	0.9300
C5—C6	1.375 (3)	C13—H13	0.9300
C6—C7	1.384 (3)	C14—H14	0.9300
C7—C8	1.369 (3)	C16—H16	0.9300
C9—C10	1.460 (3)	C17—H17	0.9300
C10—C11	1.162 (3)	C18—H18A	0.9600
C12—C13	1.385 (2)	C18—H18B	0.9600
C12—C17	1.387 (2)	C18—H18C	0.9600
C13—C14	1.384 (2)		
			
N2—N1—C4	118.01 (13)	C15—C16—C17	121.75 (17)
N1—N2—C3	126.58 (13)	C12—C17—C16	120.78 (17)
N1—N2—C9	113.85 (13)	C1—C5—H5	120.00
C3—N2—C9	119.36 (13)	C6—C5—H5	120.00
C2—C1—C4	117.79 (13)	C5—C6—H6	120.00
C2—C1—C5	117.93 (14)	C7—C6—H6	120.00
C4—C1—C5	124.26 (13)	C6—C7—H7	120.00
C1—C2—C3	119.84 (14)	C8—C7—H7	120.00
C1—C2—C8	120.52 (14)	C2—C8—H8	120.00
C3—C2—C8	119.64 (13)	C7—C8—H8	120.00
O1—C3—N2	121.00 (16)	N2—C9—H9A	109.00
O1—C3—C2	124.23 (16)	N2—C9—H9B	109.00
N2—C3—C2	114.77 (13)	C10—C9—H9A	109.00
N1—C4—C1	122.64 (13)	C10—C9—H9B	109.00
N1—C4—C12	114.62 (14)	H9A—C9—H9B	108.00
C1—C4—C12	122.73 (13)	C10—C11—H11	180.00
C1—C5—C6	120.59 (15)	C12—C13—H13	119.00
C5—C6—C7	120.79 (17)	C14—C13—H13	120.00
C6—C7—C8	119.87 (18)	C13—C14—H14	119.00
C2—C8—C7	120.25 (16)	C15—C14—H14	119.00
N2—C9—C10	112.59 (16)	C15—C16—H16	119.00
C9—C10—C11	178.6 (2)	C17—C16—H16	119.00
C4—C12—C13	121.31 (14)	C12—C17—H17	120.00
C4—C12—C17	121.03 (15)	C16—C17—H17	120.00
C13—C12—C17	117.61 (14)	C15—C18—H18A	109.00
C12—C13—C14	121.04 (16)	C15—C18—H18B	109.00
C13—C14—C15	121.49 (17)	C15—C18—H18C	110.00
C14—C15—C16	117.33 (15)	H18A—C18—H18B	109.00
C14—C15—C18	121.90 (17)	H18A—C18—H18C	109.00
C16—C15—C18	120.77 (16)	H18B—C18—H18C	109.00
			
C4—N1—N2—C3	5.4 (2)	C8—C2—C3—O1	2.6 (3)
C4—N1—N2—C9	−179.97 (16)	C8—C2—C3—N2	−176.34 (16)
N2—N1—C4—C1	0.6 (2)	C1—C2—C8—C7	−1.5 (3)
N2—N1—C4—C12	−178.36 (14)	C3—C2—C8—C7	177.55 (17)
N1—N2—C3—O1	174.12 (16)	N1—C4—C12—C13	−50.0 (2)
N1—N2—C3—C2	−6.9 (2)	N1—C4—C12—C17	127.20 (17)
C9—N2—C3—O1	−0.3 (3)	C1—C4—C12—C13	131.06 (17)
C9—N2—C3—C2	178.71 (15)	C1—C4—C12—C17	−51.7 (2)
N1—N2—C9—C10	−83.42 (18)	C1—C5—C6—C7	−0.2 (3)
C3—N2—C9—C10	91.67 (19)	C5—C6—C7—C8	1.3 (3)
C4—C1—C2—C3	2.2 (2)	C6—C7—C8—C2	−0.4 (3)
C4—C1—C2—C8	−178.72 (15)	C4—C12—C13—C14	177.07 (16)
C5—C1—C2—C3	−176.58 (15)	C17—C12—C13—C14	−0.2 (3)
C5—C1—C2—C8	2.5 (2)	C4—C12—C17—C16	−176.53 (17)
C2—C1—C4—N1	−4.1 (2)	C13—C12—C17—C16	0.8 (3)
C2—C1—C4—C12	174.76 (15)	C12—C13—C14—C15	−0.4 (3)
C5—C1—C4—N1	174.61 (16)	C13—C14—C15—C16	0.5 (3)
C5—C1—C4—C12	−6.5 (2)	C13—C14—C15—C18	−178.98 (17)
C2—C1—C5—C6	−1.6 (2)	C14—C15—C16—C17	0.1 (3)
C4—C1—C5—C6	179.67 (16)	C18—C15—C16—C17	179.55 (18)
C1—C2—C3—O1	−178.30 (17)	C15—C16—C17—C12	−0.7 (3)
C1—C2—C3—N2	2.7 (2)		

### Hydrogen-bond geometry (Å, º)

**Table:**

*D*—H···*A*	*D*—H	H···*A*	*D*···*A*	*D*—H···*A*
C6—H6···O1^i^	0.93	2.45	3.322 (2)	157

Symmetry code: (i) *x*−1/2, −*y*−1/2, *z*−1/2.
